# Supplementary material for: Degradation of Bisphenol A by Bacillus subtilis P74 Isolated from Traditional Fermented Soybean Foods
Source: Microorganisms. 2023 Aug 22;11(9):2132. doi: 10.3390/microorganisms11092132 (PMC10536603; doi:10.3390/microorganisms11092132)
Supplement: Supplementary file 1 [file microorganisms-11-02132-s001.zip › microorganisms-2544410-supplementary.pdf]

Supplementary Materials:

Degradation of Bisphenol A by *Bacillus subtilis* P74 Isolated from Traditional Fermented Soybean Foods

Young Kyoung Park and Young-Wook Chin *

Research Group of Traditional Food, Korea Food Research Institute, Iseo-myeon, Wanju-gun 55365, Jeollabuk-do, Republic of Korea

* Correspondence: ywchin@kfri.re.kr; Tel.: +82-63-219-9317

Table S1. BPA-degrading ability of microorganisms isolated from Korean traditional fermented soybean products.

Species	N[a]	BPA degradation rate (%)
Bacillus aerius	1	31.0[b]
Bacillus amyloliquefaciens	13	22.7 ± 13.1[c]<br>(7.6 – 54.0)
Bacillus atrophaeus	8	49.9 ± 7.2<br>(41.8 – 64.4)
Bacillus coagulans	2	36.9 ± 46.9<br>(3.7 – 70.1)
Bacillus glycinifermentans	2	25.4 ± 14.5<br>(15.1 – 35.7)
Bacillus licheniformis	14	43.7 ± 13.4<br>(6.2 – 54.5)
Bacillus paralicheniformis	3	14.3 ± 15.2<br>(-3.3 – 23.9)
Bacillus safensis	1	24.7
Bacillus siamensis	2	30.4 ± 4.9<br>(26.9 – 33.9)
Bacillus sonorensis	6	64.8 ± 4.8<br>(58.8 – 70.9)
Bacillus spp.	23	33.2 ± 12.2<br>(10.4 – 58.0)
Bacillus sporothermodurans	1	41.8
Bacillus subtilis	48	43.6 ± 18.3<br>(14.2 – 92.1)
Bacillus thermoamylovorans	2	29.1 ± 41.0<br>(0.0 – 58.1)
Bacillus vallismortis	1	27.9
Bacillus velezensis	1	19.9
Brevibacterium frigoritolerans	1	12.0

[a] The number of strains tested.
[b] The value obtained from a single strain.
[c] Mean ± standard deviation (the range from minimum to maximum).

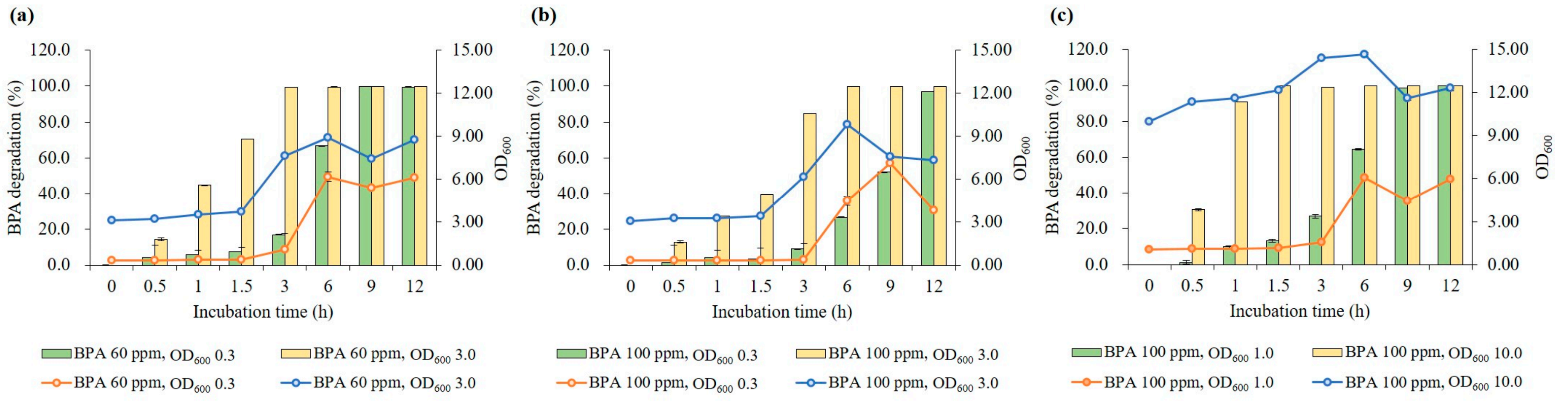

Figure S1. Performance of BPA degradation by *B. subtilis* P74 in the flask culture containing (a) 60 mg/L and (b, c) 100 mg/L BPA at incubation temperatures of 40°C. The data represent mean ± standard deviation from three measurements of harvested samples.

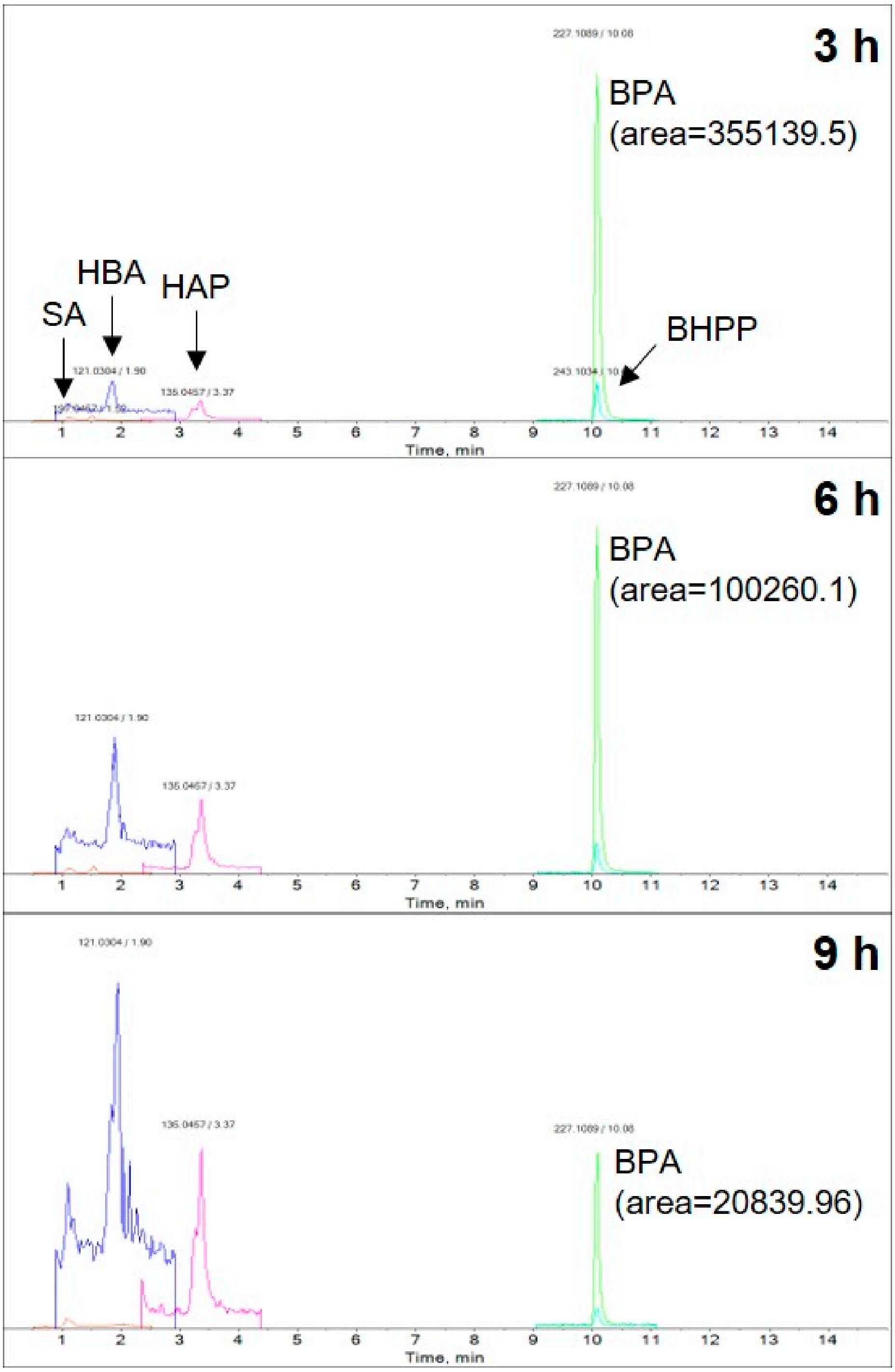

Figure S2. Ion chromatogram of the BPA degradation by *B. subtilis* P74 during the fermentation. Candidate metabolites were detected; 4-hydroxybenzaldehyde (HBA), 4-hydroxyacetophenone (HAP), syringic acid (SA), and 1,2-bis(4-hydroxyphenyl)-2-propanol (BHPP)

m/z<br>RT	Formula<br>Finder results	Candidate metabolites	MS spectrum
121.0304<br>1.90	$C_7H_6O_2$	4-Hydroxybenzaldehyde<br>(HBA)<br>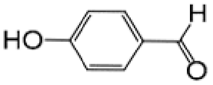	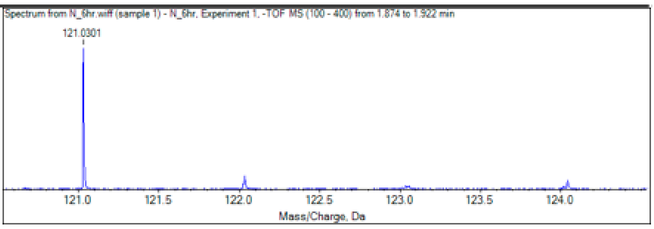
135.0457<br>3.37	$C_8H_8O_2$	4-Hydroxyacetophenone<br>(HAP)<br>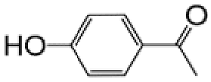	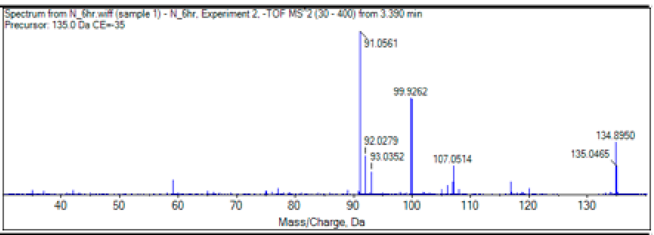
197.0457<br>1.52	$C_9H_{10}O_5$	Syringic acid<br>(SA)<br>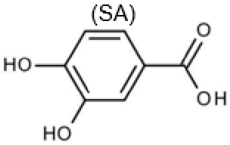	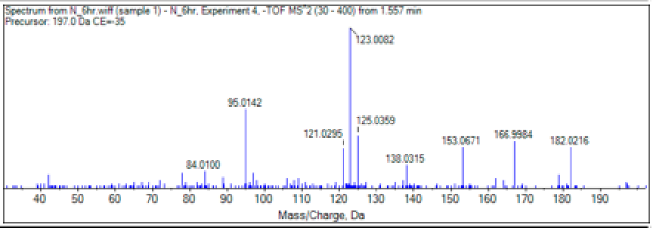
227.1089<br>10.08	$C_{15}H_{16}O_2$	Bisphenol A<br>(BPA)<br>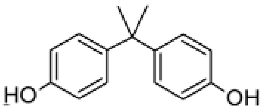	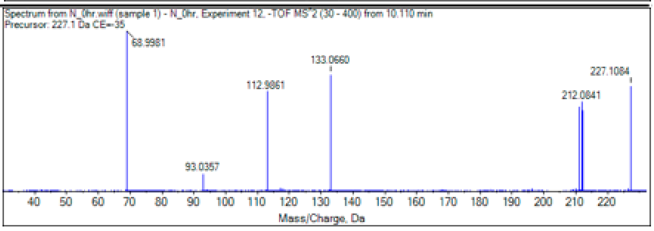
243.1034<br>10.08	$C_{15}H_{16}O_3$	1,2-Bis(4-hydroxyphenyl)-<br>2-propanol<br>(BHPP)<br>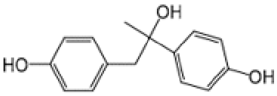	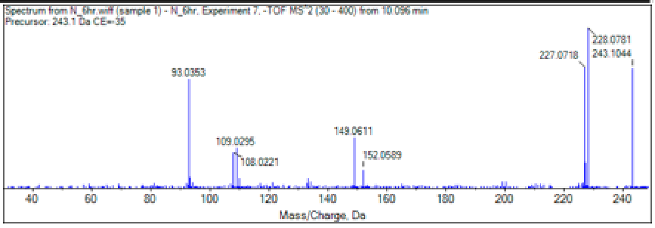

Figure S3. MS/MS spectrum and structure of the candidate metabolites found during BPA degradation by *B. subtilis* P74.
